# Gastric Epithelioid Mesenchymal Tumor with the *EWSR1::CREM* Fusion Gene: A Case Report

**DOI:** 10.70352/scrj.cr.24-0137

**Published:** 2025-04-09

**Authors:** Nao Yoshizawa, Hirokazu Yamaguchi, Taichiro Yoshimoto, Koji Uraushihara, Akihiko Yoshida

**Affiliations:** 1 Department of Gastroenterological Surgery, Showa General Hospital, Kodaira, Tokyo, Japan; 2 Department of Diagnostic Pathology, Showa General Hospital, Kodaira, Tokyo, Japan; 3 Department of Gastroenterology, Showa General Hospital, Kodaira, Tokyo, Japan; 4 Department of Diagnostic Pathology, National Cancer Center Hospital, Tokyo, Japan

**Keywords:** *EWSR1::CREM* gene fusion, *EWSR1/FUS::CREB* fusion, gastric mesenchymal tumor

## Abstract

**INTRODUCTION:**

In recent years, new molecularly defined tumor groups have been reported among tumors previously considered unclassifiable. Among them, gene fusions involving the CREB family of transcription factors, including cAMP-responsive element modulator (*CREM*), with genes encoding FET family RNA-binding proteins, such as Ewing sarcoma breakpoint region 1 (*EWSR1*), have recently been shown to be implicated in driving the pathogenesis of various tumor types. Here, we report our experience with a gastric mesenchymal tumor with epithelioid histology and an *EWSR1::CREM* fusion, which is rare but requires caution.

**CASE PRESENTATION:**

A 58-year-old man with epigastric pain underwent esophagogastroduodenoscopy, which revealed a submucosal tumor, 40 × 30 mm in size, at the greater curvature of the upper gastric body. Surgical resection was scheduled because of easy bleeding from the tumor and because biopsy could not establish a diagnosis. The tumor was clinically considered benign because there was no significant accumulation on positron emission tomography scans. Therefore, we performed a local resection of the stomach. Histologically, the tumor consisted of a proliferation of keratin-positive, relatively uniform epithelioid cells arranged in sheets, with a scattering of lymphoid follicles in the surrounding area. Based on a pathology consultation, the tumor was diagnosed as a mesenchymal tumor with *EWSR1::CREM* fusion.

**CONCLUSION:**

We experienced a gastric epithelioid mesenchymal tumor with *EWSR1::CREM* fusion genes. Since a malignant course has been reported in similar tumors in the stomach and abdominal cavity, such patients require careful follow-up.

## Abbreviations

AFH
: angiomatoid fibrous histiocytoma
CCS
: clear cell sarcoma
CCSLGT
: clear cell sarcoma-like tumors of the gastrointestinal tract
CREM
: cAMP-responsive element modulator
EWSR1
: Ewing sarcoma breakpoint region 1
EWSR1/FUS::CREB fusion
: EWSR1 or FUS and CREB family fusion
FISH
: fluorescence in situ hybridization
FUS
: fused in sarcoma
GNET
: gastrointestinal neuroectodermal tumor
LECS
: laparoscopic and endoscopic cooperative surgery

## INTRODUCTION

In recent years, the increasing use of genetic testing in routine surgical pathology practice has led to the discovery of fusion-associated neoplasms, and new molecularly defined tumor groups have been reported among tumors previously considered unclassifiable. Neoplasms with fusions between Ewing sarcoma breakpoint region 1 (*EWSR1*) or fused in sarcoma (*FUS*) genes and genes encoding the CREB family of transcription factors (ATF1, CREB1, and cAMP-responsive element modulator [CREM]) are one such tumor type. These fusions have been reported to drive the pathogenesis of various tumor types with mesenchymal, neuroectodermal, and epithelial lineages.^1)^

Most tumors in the gastrointestinal tract with EWSR1/FUS and CREB family fusions (*EWSR1/FUS::CREB* fusion) are reportedly clear cell sarcoma-like tumors of the gastrointestinal tract (CCSLGT).^2,3)^ Here, we report our experience with a gastric epithelioid mesenchymal tumor with an *EWSR1::CREM* fusion that was distinct from established entities.

## CASE PRESENTATION

A 58-year-old man with a history of occasional epigastric pain for approximately 6 months underwent a medical checkup at a local hospital. Stomach fluoroscopy revealed a tumor located at the greater curvature of the upper gastric body, for which he was referred to our hospital.

**Fig. 1:**
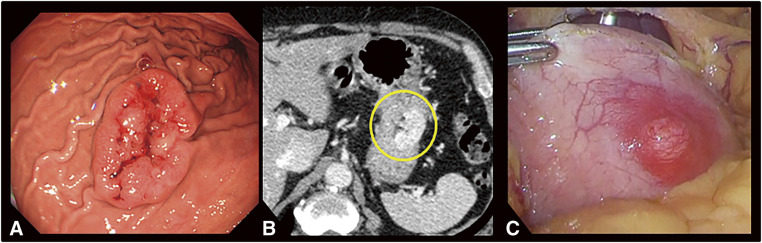
(**A**) Endoscopic findings. A protruding lesion measuring 40 × 30 mm, which had an uneven surface and bled easily, was observed along the greater curvature of the upper part of the stomach body. (**B**) CT findings. A contrast-enhanced mass (yellow circle) was observed in the upper part of the stomach body. (**C**) Laparoscopic findings. The serous surface of the tumor was smooth but appeared hyperemic. CT, computed tomography

Esophagogastroduodenoscopy showed a tumor 40 × 30 mm in size, with easy bleeding, an uneven shape, and a deep depression in the center (**Fig. 1A**). Since the entire tumor surface, including the depression, was covered by noncancerous epithelium, it was diagnosed as a gastric submucosal tumor. Hematoxylin–eosin staining of biopsy specimens showed a spindle cell tumor. However, no definitive diagnosis could be made despite using various types of immunostains. Endoscopic ultrasonography showed a uniform isoechoic mass that appeared to be derived from the fourth layer (i.e., the muscularis propria). A contrast computed tomography scan revealed a contrast-enhanced mass in the upper part of the stomach body (**Fig. 1B**), with neither invasion of the peripheral organs nor distant metastases. Positron emission tomography showed only mild accumulation in the gastric tumor, with no accumulation in other areas.

Based on these results, we considered the possibility of malignancy to be low, but since the tumor bled very easily and could not be definitively diagnosed by biopsy, we decided to perform local gastric resection. Laparoscopic evaluation showed that the serous surface of the tumor was smooth but appeared hyperemic (**Fig. 1C**). The technique of laparoscopic and endoscopic cooperative surgery (LECS) was used to perform local gastrectomy. Due to the relatively large size of the tumor, the procedure was performed using inverted LECS with Crown methods,^4)^ which were designed to prevent the outflow of gastric contents. After excising the lesion, the open ends of the stomach wound were aligned and closed with a linear stapler. The surgical time was 182 min, and estimated blood loss was below measurable levels. The patient’s postoperative course was uneventful, and he was discharged 7 days after surgery. Currently, 1 and a half years after surgery, he remains alive without recurrence.

**Fig. 2:**
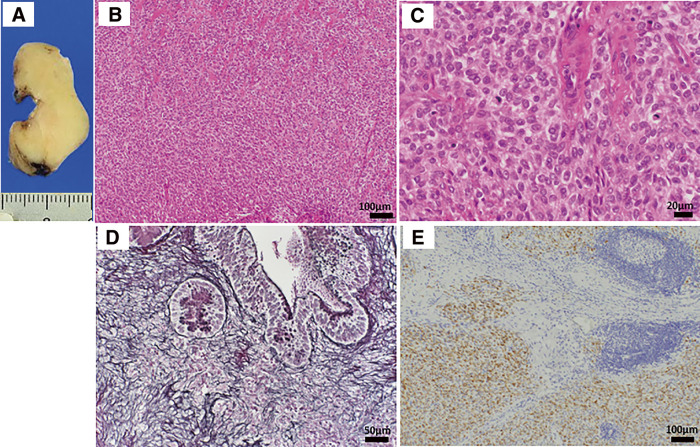
(**A**) Cut surface of the tumor. The tumor consisted of a solid lesion that was shiny and yellowish-white in color. (**B**) H&E staining with ×10 magnification showed relatively uniform epithelioid cells with sheet-like proliferation and intervening hyalinized eosinophilic stroma. (**C**) H&E staining (×40 magnification). The tumor cells had a relatively uniform round-to-oval nucleus and scant eosinophilic cytoplasm. (**D**) Silver impregnation staining (×20 magnification). Argyrophil fibers were present between the tumor cells. (**E**) Immunohistochemical findings (×10 magnification). AE1/AE3 was partially weakly positive. Lymphoid follicles were observed at the periphery of the tumor. H&E, hematoxylin–eosin

Pathological evaluation of the specimen showed that the tumor, measuring 4.5 × 2.7 × 1.6 cm, was slightly shiny and yellowish-white in color (**Fig. 2A**). Histopathological assessment revealed relatively homogeneous epithelioid cells extending from the mucosal lamina propria to the muscularis propria and slightly to the subserosal layer, with the cells proliferating in sheets with intervening hyalinized stroma (**Fig. 2B**). Scattered lymphoid follicles were observed at the periphery of the tumor. The tumor cells had relatively uniform nuclei with a round to oval shape and scant eosinophilic cytoplasm (**Fig. 2C**). The mitotic count was 3/10 high-power fields, and the cell boundaries were indistinct (**Fig. 2C**). Silver impregnation staining showed the presence of argyrophil fibers between the tumor cells, suggesting that it was a mesenchymal tumor (**Fig. 2D**). The surgical margin was negative.

**Fig. 3:**
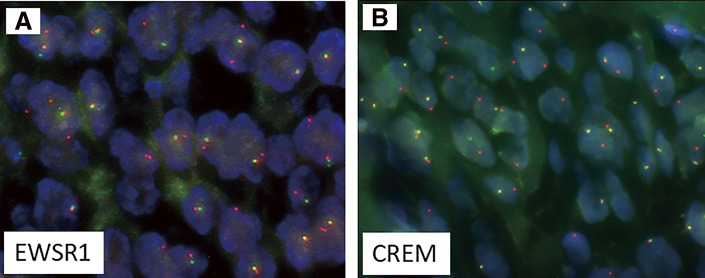
*EWSR1::CREM* fusion was identified using FISH. The tumor showed *EWSR1* (A) and *CREM* (B) rearrangements. The isolated orange signals indicate gene rearrangements. *CREM*, cAMP-responsive element modulator; *EWSR1*, Ewing sarcoma breakpoint region 1; FISH, fluorescence in situ hybridization

The tumor cells were immunohistochemically positive for D2-40 (diffuse), α-smooth muscle actin (diffuse), Bcl-2 (diffuse), AE1/AE3 (focal) (**Fig. 2E**), and Kit (focal), and negative for DOG1, desmin, S100, CD34, EMA, *β*-catenin, ALK, CD21, LCA, and CD68. The Ki-67 proliferation index was approximately 7%. Fluorescence in situ hybridization (FISH) assays showed negative evidence of *SS18* rearrangement. Gastrointestinal stromal tumor, solitary fibrous tumor, smooth muscle tumor, desmoid fibromatosis, inflammatory myofibroblastic tumor, follicular dendritic cell sarcoma, and synovial sarcoma were included in the differential diagnosis, but the findings were not typical for any of these diseases. Subsequently, at a pathology consultation, the tumor was found to be negative for MUC4 and SSX (C-term), while FISH analysis detected rearrangements for both *EWSR1* and *CREM* genes (**Fig. 3**). Based on these findings, the tumor was diagnosed as an epithelioid mesenchymal tumor with *EWSR1::CREM* gene fusion.

## DISCUSSION

*EWSR1::CREM* fusion is one of the fusions between *EWSR1* or *FUS* and the CREB family of genes that have been reported in various tumor entities since approximately 2017.^5)^ Types of tumors with these fusions include angiomatoid fibrous histiocytoma (AFH), soft tissue and gastrointestinal clear cell sarcoma (CCS), primary pulmonary myxoid sarcoma, hyalinizing clear cell carcinoma of the salivary gland, and malignant mesotheliomas.^1)^ EWSR1, along with FUS, belongs to the FET family of RNA-binding proteins.^6)^ EWSR1 is involved in the crucial regulation of proper centromere function and inheritance in interphase cells.^7)^ Meanwhile, CREM, together with ATF1 and CREB1, belongs to the group of transcription factors called CREB family transcription factors.^1,2)^ Among these transcription factors, CREM is a newly recognized member in human tumors, and the phenotypic spectrum associated with CREM fusion is still being clarified.^2)^

Most gastric tumors with *EWSR1/FUS::CREB* fusions have been reported to be CCSLGT, also known as gastrointestinal neuroectodermal tumors (GNETs). The tumor in our patient had histological findings distinct from GNETs but was similar to 3 previously reported tumors,^1,8,9)^ which are summarized in **Table 1**. The 4 patients, including the present case, ranged in age from 25 to 64 years and consisted of 3 men and 1 woman. Although interpreted as mesenchymal tumors, cytokeratin or EMA positivity was observed in 3 of the cases, including our patient. In case 3, which showed a mixture of epithelial and mesenchymal components in gastritis cystica profunda, the disease course was benign, while the other 2 patients experienced metastasis and recurrence. Among these 2 cases, liver metastasis occurred 1 year after diagnosis in the first case, although the patient was subsequently lost to follow-up. The second case underwent resection of the initial gastric lesion and a recurrent lesion, followed by treatment with imatinib, sunitinib, and radiation therapy for the recurrence.

**Table 1:** Literature review of cases of gastric epithelioid mesenchymal tumors with *EWSR1/FUS::CREB* fusion

Case	Age	Sex	Primary site	Size (cm)	Metastasis		IHC-positive Epithelial markers	Fusion	Outcome (mo)	References
1	25	M	Intra-abdominal continuing from fornix of the stomach	7	+	Liver	EMA	*EWSR1::CREM*	Rec (12) AWD (12)	^1)^
2	32	M	Stomach	3	+	Liver Peritoneum Lung Spleen	AE1/AE3	*EWSR1::CREB1*	Rec (48, 56) AWD (122)	^8)^
3	64	F	Stomach	8	−			*EWSR1::CREM*	NED (28)	^9)^
Our case	58	M	Stomach	4.5	−		AE1/AE3	*EWSR1::CREM*	NED (20)	

AWD, alive with disease; *CREM*, cAMP-responsive element modulator; *EWSR1*, Ewing sarcoma breakpoint region 1; *EWSR1/FUS::CREB* fusion, EWSR1 or FUS and CREB family fusion; F, female; IHC, immunohistochemistry; M, male; mo, months; NED, alive with no evidence of disease; rec, recurrence.

In a broader context, similar epithelioid mesenchymal tumors with keratin expression and *EWSR1/FUS::CREB* fusion have been reported in the abdominal cavity outside the stomach, with many of these tumors showing malignant behavior, including recurrence, metastasis, and resistance to chemotherapy.^1,8)^ Considering these previous tumor reports, we believe that our case should be carefully followed up for potential malignancy. There are still very few reports of gastric epithelioid mesenchymal tumors with *EWSR1/FUS::CREB* fusion that do not fit the criteria for GNETs, and the collection of additional cases is needed to better understand their epidemiology, symptoms, prognosis, and treatment.

## CONCLUSION

We experienced a case of gastric epithelioid mesenchymal tumor with *EWSR1::CREM* fusion genes that did not fit the diagnostic criteria of established tumor entities. Since a malignant course has been previously reported in the literature for similar tumors, careful follow-up is required for these cases.

## ACKNOWLEDGMENTS

The authors thank FORTE Science Communications (https://www.forte-science.co.jp/) for English language editing.

## DECLARATIONS

### Funding

This study received no specific grants from funding agencies in the public, commercial, or not-for-profit sectors.

### Authors’contributions

NY drafted the manuscript.

All other authors critically reviewed the manuscript.

NY, HY, and KU performed operations.

KU performed endoscopy and was responsible for preoperative diagnosis.

TY and AY carried out pathological diagnosis.

All authors approved the final manuscript and agreed to take responsibility for all aspects of the study.

### Availability of data and materials

Data presented in this case report are available from the corresponding author upon reasonable request.

### Ethics approval and consent to participate

This work does not require ethical considerations or approval.

### Consent for publication

Written informed consent was obtained from the patient for the publication of this case report and accompanying images.

### Competing interests

The authors declare that they have no competing interests.
